# 4′-Methyl-14′,19′-dioxa-4′-aza­spiro­[acenaphthyl­ene-1,5′-tetra­cyclo­[18.4.0.0^2,6^.0^8,13^]tetra­cosa­ne]-1′(24′),8′,10′,12′,20′,22′-hexa­ene-2,7′(1*H*)-dione

**DOI:** 10.1107/S1600536812046144

**Published:** 2012-11-14

**Authors:** Sibi Narayanan, Thothadri Srinivasan, Santhanagopalan Purushothaman, Raghavachary Raghunathan, Devadasan Velmurugan

**Affiliations:** a Centre of Advanced Study in Crystallography and Biophysics, University of Madras, Guindy Campus, Chennai 600 025, India; b Department of Organic Chemistry, University of Madras, Guindy Campus, Chennai 600 025, India

## Abstract

In the title compound, C_33_H_29_NO_4_, the acenaphthyl­ene ring system is essentially planar (r.m.s. deviation = 0.0290 Å). The pyrrolidine ring adopts a C-envelope conformation with a C atom displaced by 0.671 (2) Å from the mean-plane formed by the remaining ring atoms. The pyrrolidine ring is fused to acenaphthyl­ene ring system making a dihedral angle of 88.0 (7)°. In the crystal, mol­ecules are linked into *R* ^2^_2_(9) dimers *via* C—H⋯N and C—H⋯O hydrogen bonds. Two C atoms act as donors to the same O atom acceptor, resulting in the formation of *R* ^2^_1_(7) ring motifs. These two motifs combine to form hydrogen-bonded sheets running along the *a-* and *b*-axis directions.

## Related literature

For background to natural and synthetic pharmacologically active pyrrolidines, see: Waldmann (1995 ▶). For related structures, see: Augustine *et al.* (2010 ▶); Narayanan *et al.* (2012 ▶). For graph-set motifs, see: Bernstein *et al.* (1995 ▶).
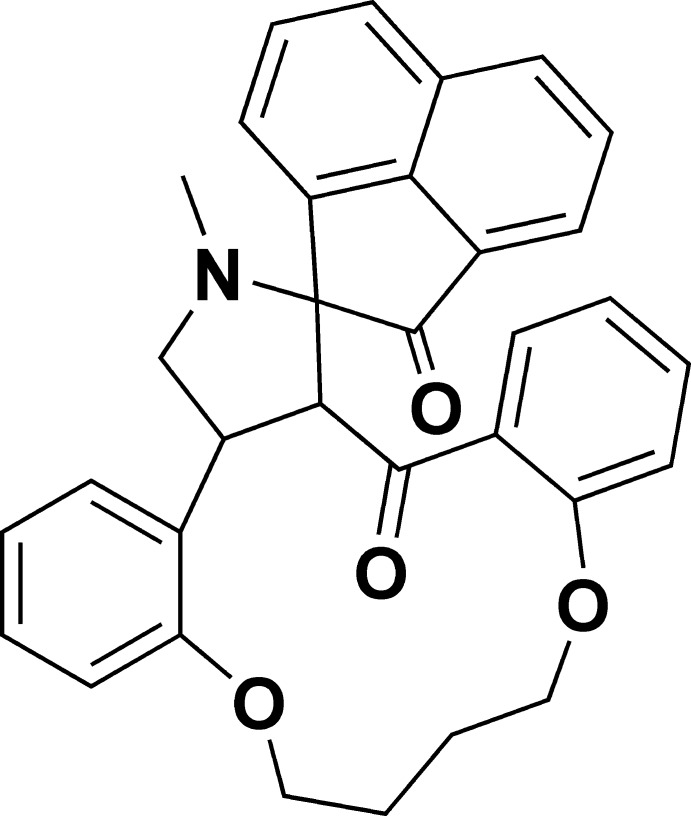


## Experimental

### 

#### Crystal data

C_33_H_29_NO_4_
*M* *_r_* = 503.57
Monoclinic, 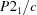

*a* = 11.248 (2) Å
*b* = 16.609 (3) Å
*c* = 14.037 (3) Å
β = 92.965 (6)°
*V* = 2618.8 (9) Å^3^
*Z* = 4
Mo *K*α radiation
μ = 0.08 mm^−1^
*T* = 293 K
0.25 × 0.22 × 0.19 mm

#### Data collection

Bruker APEXII CCD area-detector diffractometer
Absorption correction: multi-scan (*SADABS*; Bruker, 2008 ▶) *T*_min_ = 0.979, *T*_max_ = 0.984
24740 measured reflections
6363 independent reflections
4183 reflections with *I* > 2σ(*I*)
*R*_int_ = 0.035

#### Refinement

*R*[*F* ^2^ > 2σ(*F* ^2^)] = 0.045
*wR*(*F* ^2^) = 0.128
*S* = 1.01
6363 reflections
345 parameters
H-atom parameters constrained
Δρ_max_ = 0.22 e Å^−3^
Δρ_min_ = −0.21 e Å^−3^

### 

Data collection: *APEX2* (Bruker, 2008 ▶); cell refinement: *SAINT* (Bruker, 2008 ▶); data reduction: *SAINT*; program(s) used to solve structure: *SHELXS97* (Sheldrick, 2008 ▶); program(s) used to refine structure: *SHELXL97* (Sheldrick, 2008 ▶); molecular graphics: *ORTEP-3* (Farrugia, 2012 ▶); software used to prepare material for publication: *SHELXL97* and *PLATON* (Spek, 2009 ▶).

## Supplementary Material

Crystal structure: contains datablock(s) global, I. DOI: 10.1107/S1600536812046144/pv2602sup1.cif

Structure factors: contains datablock(s) I. DOI: 10.1107/S1600536812046144/pv2602Isup2.hkl

Additional supplementary materials:  crystallographic information; 3D view; checkCIF report

## Figures and Tables

**Table 1:** Hydrogen-bond geometry (Å, °)

*D*—H⋯*A*	*D*—H	H⋯*A*	*D*⋯*A*	*D*—H⋯*A*
C9—H9⋯N1^i^	0.93	2.62	3.535 (3)	167
C15—H15⋯O1^i^	0.93	2.50	3.414 (2)	168
C27—H27*B*⋯O2^ii^	0.97	2.48	3.403 (2)	158
C29—H29⋯O2^ii^	0.93	2.57	3.450 (2)	159

Symmetry codes: (i) 
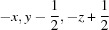
; (ii) 
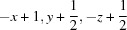
.

**Fig. 1.**
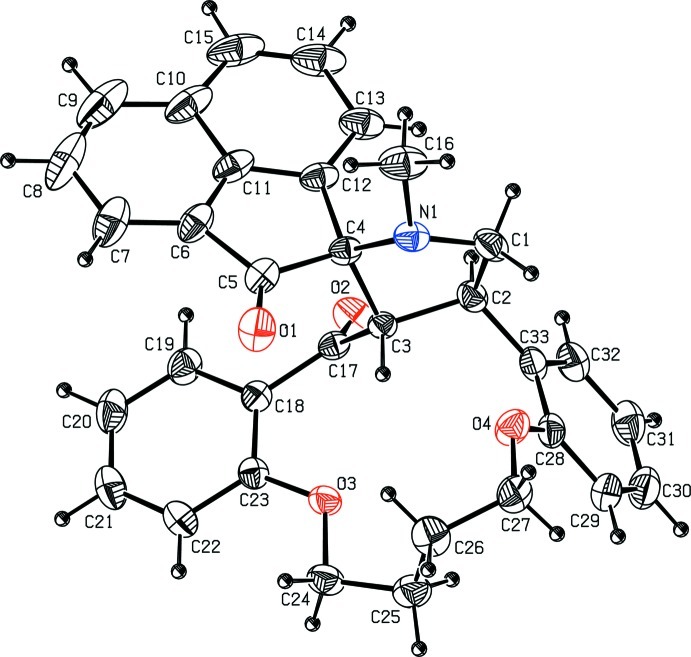
The molecular structure of the title compound, showing displacement ellipsoids drawn at the 30% probability level. H atoms are presented as small spheres of arbitrary radius.

**Fig. 2.**
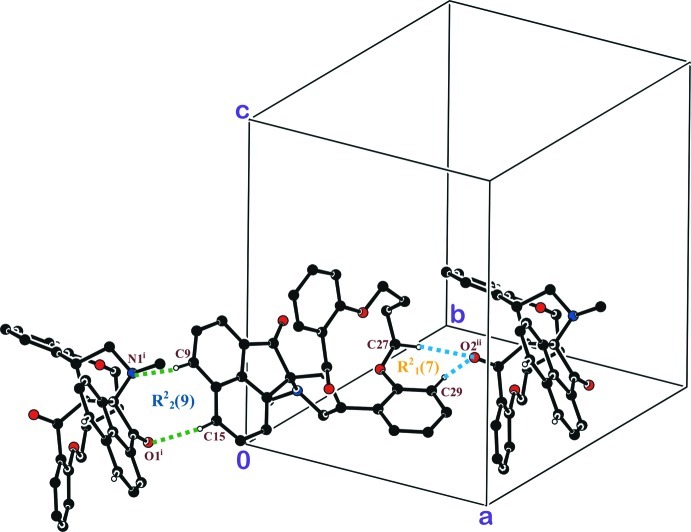
The crystal structure showing C—H···N and C—H···O hydrogen bonds (dashed lines); H atoms not involved in hydrogen bonding have been omitted for clarity. Symmetry codes: i - x, - 1/2 + y, 1/2 - z; ii 1 - x, 1/2 + y, 1/2 - z.

**Fig. 3.**
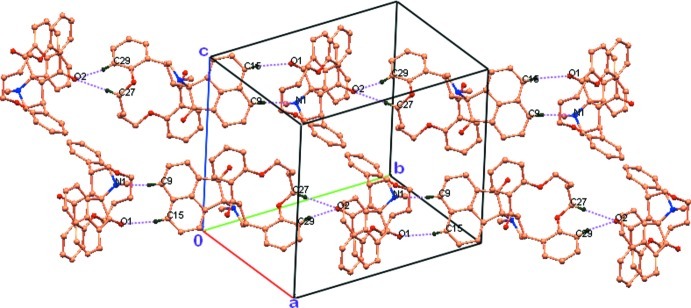
Molecular packing of the title compound, showing hydrogen bonds resulting in molecular ribbons running along the a and the b axes. H atoms not involved in hydrogen bonds have been omitted for clarity.

## supplementary crystallographic information

### Comment

Highly functionalized pyrrolidines have gained much interest in the past few years as they constitute the main structural element of many natural and synthetic pharmacologically active compounds (Waldmann, 1995). In continuation of our work on the crystal structure analysis of spiro-pyrrolidine derivatives (Narayanan *et al.*, 2012), the crystal structure of the title compound has been carried out and the results are presented here.

The bond lengths and angles in the title molecule (Fig. 1) are within normal ranges and comparable to those found in closely related structures (Narayanan *et al.*, 2012; Augustine *et al.*, 2010). The acenaphthylene ring system (C4–C15) is essentially planar (rmsd 0.0290 Å). The pyrrolidine ring (C1–C4/N1) adopts a C4-envelop conformation with C4 0.671 (2) Å displaced from the mean-plane formed by the remaining ring atoms. The pyrrolidine ring is fused to acenaphthylene ring system; the dihedral angle between these two ring systems being 88.0 (7)°.

The molecules are linked into dimers *via* C9—H9···N1 and C15—H15···O1 hydrogen bonds with the graph-set motif *R*^2^_2_(9) (Bernstein *et al.*, 1995). Similarly, atoms C27 and C29 act as donors to form bifurcated hydrogen bonds with atom O2 as an acceptor, resulting in the formation of *R*^2^_1_(7) ring motif. These two motifs combine to form a hydrogen-bonded molecular ribbons running along the *a* and *b*-axes.

### Experimental

A mixture of acenaphthylene-1,2-dione (182 mg, 1 mmol), sarcosine (90 mg, 1 mmol) and (4E)-12,17-dioxatricyclo[16.4.0.0^6,11^]docosa -1(22),4,6,8,10,18,20-heptaen-3-one (300 mg 1.0 mmol) in toluene (20 ml) was refluxed under Dean-Stark reaction condition until the disappearance of starting materials as evidenced by TLC. The reaction mixture was concentrated *in vacuo* and extracted with water (50 ml) and dichloromethane (2x50 ml). The organic layer was washed with brine solution, dried with anhydrous sodium sulfate and concentrated *in vacuo*. The residue was purified by column chromatography with hexane-ethylacetate (9:1) mixture to yield macrocycle in good yields. The product was dissolved in chloroform and heated for two minutes. The resulting solution was subjected to crystallization by slow evaporation of the solvent resulting in single crystals suitable for XRD studies.

### Refinement

All H atoms were fixed geometrically and allowed to ride on their parent C atoms, with C—H distances fixed in the range 0.93–0.98 Å with *U*_iso_(H) = 1.5*U*_eq_(C) for methyl H 1.2*U*_eq_(C) for other H atoms.

### Crystal data

**Table:**

C_33_H_29_NO_4_	*F*(000) = 1064
*M**_r_* = 503.57	*D*_x_ = 1.277 Mg m^−^^3^
Monoclinic, *P*2_1_/*c*	Mo *K*α radiation, λ = 0.71073 Å
Hall symbol: -P 2ybc	Cell parameters from 6363 reflections
*a* = 11.248 (2) Å	θ = 1.8–28.3°
*b* = 16.609 (3) Å	µ = 0.08 mm^−^^1^
*c* = 14.037 (3) Å	*T* = 293 K
β = 92.965 (6)°	Block, colorless
*V* = 2618.8 (9) Å^3^	0.25 × 0.22 × 0.19 mm
*Z* = 4	

### Data collection

**Table:**

Bruker APEXII CCD area-detector diffractometer	6363 independent reflections
Radiation source: fine-focus sealed tube	4183 reflections with *I* > 2σ(*I*)
Graphite monochromator	*R*_int_ = 0.035
ω and φ scans	θ_max_ = 28.3°, θ_min_ = 1.8°
Absorption correction: multi-scan (*SADABS*; Bruker, 2008)	*h* = −14→14
*T*_min_ = 0.979, *T*_max_ = 0.984	*k* = −21→19
24740 measured reflections	*l* = −18→18

### Refinement

**Table:**

Refinement on *F*^2^	Secondary atom site location: difference Fourier map
Least-squares matrix: full	Hydrogen site location: inferred from neighbouring sites
*R*[*F*^2^ > 2σ(*F*^2^)] = 0.045	H-atom parameters constrained
*wR*(*F*^2^) = 0.128	*w* = 1/[σ^2^(*F*_o_^2^) + (0.0523*P*)^2^ + 0.572*P*] where *P* = (*F*_o_^2^ + 2*F*_c_^2^)/3
*S* = 1.01	(Δ/σ)_max_ < 0.001
6363 reflections	Δρ_max_ = 0.22 e Å^−^^3^
345 parameters	Δρ_min_ = −0.21 e Å^−^^3^
0 restraints	Extinction correction: *SHELXL97* (Sheldrick, 2008), Fc^*^=kFc[1+0.001xFc^2^λ^3^/sin(2θ)]^-1/4^
Primary atom site location: structure-invariant direct methods	Extinction coefficient: 0.0125 (10)

### Special details

**Table:**

Geometry. All e.s.d.'s (except the e.s.d. in the dihedral angle between two l.s. planes) are estimated using the full covariance matrix. The cell e.s.d.'s are taken into account individually in the estimation of e.s.d.'s in distances, angles and torsion angles; correlations between e.s.d.'s in cell parameters are only used when they are defined by crystal symmetry. An approximate (isotropic) treatment of cell e.s.d.'s is used for estimating e.s.d.'s involving l.s. planes.
Refinement. Refinement of *F*^2^ against ALL reflections. The weighted *R*-factor *wR* and goodness of fit *S* are based on *F*^2^, conventional *R*-factors *R* are based on *F*, with *F* set to zero for negative *F*^2^. The threshold expression of *F*^2^ > σ(*F*^2^) is used only for calculating *R*-factors(gt) *etc*. and is not relevant to the choice of reflections for refinement. *R*-factors based on *F*^2^ are statistically about twice as large as those based on *F*, and *R*- factors based on ALL data will be even larger.

### Fractional atomic coordinates and isotropic or equivalent isotropic displacement parameters (Å^2^)

**Table:**

	*x*	*y*	*z*	*U*_iso_*/*U*_eq_	
C1	0.23872 (16)	0.09798 (10)	0.09848 (11)	0.0548 (4)	
H1A	0.2555	0.1542	0.0861	0.066*	
H1B	0.2240	0.0706	0.0380	0.066*	
C2	0.34391 (14)	0.05856 (9)	0.15621 (10)	0.0447 (4)	
H2	0.3567	0.0056	0.1276	0.054*	
C3	0.29234 (12)	0.04354 (8)	0.25405 (9)	0.0358 (3)	
H3	0.2970	0.0936	0.2911	0.043*	
C4	0.16007 (13)	0.02549 (8)	0.22597 (10)	0.0407 (3)	
C5	0.08158 (14)	0.03292 (9)	0.31428 (12)	0.0487 (4)	
C6	0.02699 (14)	−0.04693 (10)	0.32978 (14)	0.0580 (5)	
C7	−0.04217 (18)	−0.07611 (14)	0.39953 (19)	0.0829 (7)	
H7	−0.0639	−0.0439	0.4499	0.100*	
C8	−0.0792 (2)	−0.15770 (18)	0.3916 (3)	0.1069 (10)	
H8	−0.1244	−0.1792	0.4391	0.128*	
C9	−0.0513 (2)	−0.20604 (16)	0.3171 (3)	0.1046 (10)	
H9	−0.0800	−0.2585	0.3142	0.126*	
C10	0.02022 (18)	−0.17780 (11)	0.24486 (19)	0.0766 (6)	
C11	0.05975 (15)	−0.09741 (10)	0.25487 (14)	0.0569 (5)	
C12	0.13465 (14)	−0.06061 (9)	0.19125 (12)	0.0488 (4)	
C13	0.16914 (19)	−0.10407 (10)	0.11489 (14)	0.0652 (5)	
H13	0.2189	−0.0812	0.0714	0.078*	
C14	0.1285 (2)	−0.18403 (12)	0.10263 (18)	0.0841 (7)	
H14	0.1515	−0.2128	0.0498	0.101*	
C15	0.0580 (2)	−0.22028 (12)	0.1642 (2)	0.0908 (8)	
H15	0.0340	−0.2733	0.1538	0.109*	
C16	0.02011 (19)	0.08788 (12)	0.10598 (16)	0.0748 (6)	
H16A	0.0151	0.0405	0.0667	0.112*	
H16B	0.0105	0.1349	0.0665	0.112*	
H16C	−0.0415	0.0865	0.1508	0.112*	
C17	0.35059 (13)	−0.02308 (8)	0.31245 (10)	0.0392 (3)	
C18	0.33202 (13)	−0.02667 (8)	0.41733 (10)	0.0402 (3)	
C19	0.29524 (16)	−0.09961 (10)	0.45511 (13)	0.0562 (4)	
H19	0.2801	−0.1431	0.4146	0.067*	
C20	0.28089 (18)	−0.10834 (12)	0.55164 (15)	0.0695 (5)	
H20	0.2542	−0.1569	0.5757	0.083*	
C21	0.30611 (18)	−0.04520 (13)	0.61134 (13)	0.0671 (5)	
H21	0.2985	−0.0515	0.6766	0.081*	
C22	0.34253 (16)	0.02753 (11)	0.57657 (12)	0.0561 (4)	
H22	0.3598	0.0699	0.6183	0.067*	
C23	0.35360 (13)	0.03799 (9)	0.47915 (10)	0.0413 (3)	
C24	0.41564 (17)	0.17608 (10)	0.50022 (12)	0.0553 (4)	
H24A	0.4800	0.1637	0.5465	0.066*	
H24B	0.3459	0.1909	0.5341	0.066*	
C25	0.45091 (18)	0.24358 (11)	0.43577 (13)	0.0621 (5)	
H25A	0.5106	0.2239	0.3941	0.075*	
H25B	0.4868	0.2863	0.4745	0.075*	
C26	0.34744 (18)	0.27817 (11)	0.37493 (13)	0.0642 (5)	
H26A	0.2802	0.2419	0.3776	0.077*	
H26B	0.3244	0.3291	0.4023	0.077*	
C27	0.37315 (18)	0.29175 (9)	0.27161 (13)	0.0589 (5)	
H27A	0.3124	0.3262	0.2417	0.071*	
H27B	0.4495	0.3184	0.2676	0.071*	
C28	0.47683 (15)	0.18063 (9)	0.19420 (11)	0.0476 (4)	
C29	0.58811 (16)	0.21697 (11)	0.19675 (13)	0.0592 (5)	
H29	0.5977	0.2688	0.2210	0.071*	
C30	0.68444 (17)	0.17641 (13)	0.16345 (14)	0.0694 (5)	
H30	0.7587	0.2011	0.1655	0.083*	
C31	0.67185 (18)	0.10011 (14)	0.12739 (14)	0.0694 (5)	
H31	0.7370	0.0731	0.1046	0.083*	
C32	0.56117 (17)	0.06348 (12)	0.12523 (12)	0.0591 (5)	
H32	0.5531	0.0116	0.1009	0.071*	
C33	0.46186 (15)	0.10206 (10)	0.15840 (10)	0.0468 (4)	
N1	0.13583 (12)	0.09043 (7)	0.15743 (9)	0.0472 (3)	
O1	0.07071 (12)	0.09415 (7)	0.35944 (9)	0.0664 (4)	
O2	0.40539 (11)	−0.07668 (7)	0.27515 (8)	0.0599 (3)	
O3	0.39011 (10)	0.10797 (6)	0.43954 (7)	0.0497 (3)	
O4	0.37498 (10)	0.21578 (7)	0.22268 (8)	0.0569 (3)	

### Atomic displacement parameters (Å^2^)

**Table:**

	*U*^11^	*U*^22^	*U*^33^	*U*^12^	*U*^13^	*U*^23^
C1	0.0767 (12)	0.0497 (9)	0.0371 (8)	0.0005 (8)	−0.0065 (8)	0.0038 (7)
C2	0.0589 (10)	0.0389 (8)	0.0367 (7)	0.0005 (7)	0.0053 (7)	−0.0021 (6)
C3	0.0430 (8)	0.0286 (7)	0.0356 (7)	−0.0007 (5)	0.0004 (6)	−0.0025 (5)
C4	0.0445 (8)	0.0300 (7)	0.0467 (8)	0.0009 (6)	−0.0044 (6)	−0.0016 (6)
C5	0.0404 (8)	0.0408 (8)	0.0647 (10)	0.0051 (6)	0.0028 (7)	0.0039 (8)
C6	0.0360 (8)	0.0512 (10)	0.0865 (13)	−0.0024 (7)	−0.0001 (8)	0.0147 (9)
C7	0.0502 (11)	0.0831 (15)	0.1170 (18)	−0.0046 (10)	0.0184 (11)	0.0275 (13)
C8	0.0591 (14)	0.0902 (19)	0.172 (3)	−0.0192 (13)	0.0140 (16)	0.057 (2)
C9	0.0675 (15)	0.0611 (15)	0.183 (3)	−0.0210 (12)	−0.0202 (17)	0.0360 (17)
C10	0.0598 (12)	0.0417 (10)	0.1246 (19)	−0.0126 (9)	−0.0300 (12)	0.0176 (12)
C11	0.0439 (9)	0.0383 (9)	0.0863 (13)	−0.0032 (7)	−0.0180 (9)	0.0071 (8)
C12	0.0512 (9)	0.0337 (8)	0.0596 (10)	−0.0002 (7)	−0.0158 (7)	−0.0022 (7)
C13	0.0867 (14)	0.0409 (9)	0.0656 (11)	0.0041 (9)	−0.0182 (10)	−0.0117 (8)
C14	0.1166 (19)	0.0401 (11)	0.0908 (15)	0.0066 (11)	−0.0411 (14)	−0.0164 (11)
C15	0.1038 (18)	0.0339 (10)	0.128 (2)	−0.0062 (11)	−0.0552 (16)	−0.0039 (13)
C16	0.0739 (13)	0.0638 (12)	0.0828 (14)	0.0077 (10)	−0.0346 (11)	−0.0002 (10)
C17	0.0404 (8)	0.0318 (7)	0.0451 (8)	−0.0005 (6)	−0.0007 (6)	−0.0023 (6)
C18	0.0384 (8)	0.0376 (8)	0.0442 (8)	0.0039 (6)	−0.0023 (6)	0.0070 (6)
C19	0.0613 (11)	0.0435 (9)	0.0634 (11)	−0.0025 (8)	−0.0003 (8)	0.0111 (8)
C20	0.0752 (13)	0.0629 (12)	0.0709 (13)	−0.0046 (10)	0.0097 (10)	0.0294 (10)
C21	0.0765 (13)	0.0773 (13)	0.0482 (10)	0.0074 (10)	0.0096 (9)	0.0210 (10)
C22	0.0633 (11)	0.0628 (11)	0.0420 (9)	0.0085 (8)	0.0010 (8)	0.0034 (8)
C23	0.0405 (8)	0.0422 (8)	0.0410 (8)	0.0047 (6)	−0.0005 (6)	0.0050 (6)
C24	0.0724 (11)	0.0472 (9)	0.0457 (9)	−0.0012 (8)	−0.0037 (8)	−0.0094 (7)
C25	0.0727 (12)	0.0509 (10)	0.0621 (11)	−0.0082 (9)	−0.0020 (9)	−0.0078 (8)
C26	0.0783 (13)	0.0507 (10)	0.0644 (11)	0.0104 (9)	0.0099 (10)	−0.0038 (8)
C27	0.0776 (12)	0.0345 (8)	0.0648 (11)	−0.0015 (8)	0.0075 (9)	0.0031 (8)
C28	0.0563 (10)	0.0461 (9)	0.0410 (8)	−0.0045 (7)	0.0083 (7)	0.0086 (7)
C29	0.0634 (11)	0.0561 (10)	0.0585 (10)	−0.0112 (9)	0.0089 (8)	0.0099 (8)
C30	0.0549 (11)	0.0847 (15)	0.0694 (12)	−0.0108 (10)	0.0103 (9)	0.0195 (11)
C31	0.0581 (12)	0.0888 (15)	0.0631 (11)	0.0089 (10)	0.0203 (9)	0.0109 (11)
C32	0.0668 (12)	0.0640 (11)	0.0477 (9)	0.0056 (9)	0.0145 (8)	0.0003 (8)
C33	0.0557 (9)	0.0500 (9)	0.0356 (7)	−0.0009 (7)	0.0103 (7)	0.0051 (6)
N1	0.0544 (8)	0.0381 (7)	0.0474 (7)	0.0035 (6)	−0.0124 (6)	0.0029 (5)
O1	0.0762 (9)	0.0486 (7)	0.0767 (8)	0.0104 (6)	0.0253 (7)	−0.0038 (6)
O2	0.0757 (8)	0.0466 (7)	0.0571 (7)	0.0223 (6)	0.0017 (6)	−0.0050 (5)
O3	0.0681 (7)	0.0412 (6)	0.0393 (5)	−0.0081 (5)	−0.0009 (5)	−0.0042 (4)
O4	0.0603 (7)	0.0441 (6)	0.0676 (7)	−0.0071 (5)	0.0152 (6)	−0.0077 (5)

### Geometric parameters (Å, º)

**Table:**

C1—N1	1.462 (2)	C17—C18	1.499 (2)
C1—C2	1.545 (2)	C18—C19	1.394 (2)
C1—H1A	0.9700	C18—C23	1.394 (2)
C1—H1B	0.9700	C19—C20	1.381 (3)
C2—C33	1.510 (2)	C19—H19	0.9300
C2—C3	1.5386 (19)	C20—C21	1.363 (3)
C2—H2	0.9800	C20—H20	0.9300
C3—C17	1.5067 (19)	C21—C22	1.373 (3)
C3—C4	1.548 (2)	C21—H21	0.9300
C3—H3	0.9800	C22—C23	1.390 (2)
C4—N1	1.4616 (18)	C22—H22	0.9300
C4—C12	1.533 (2)	C23—O3	1.3609 (17)
C4—C5	1.564 (2)	C24—O3	1.4360 (18)
C5—O1	1.2079 (19)	C24—C25	1.507 (2)
C5—C6	1.482 (2)	C24—H24A	0.9700
C6—C7	1.370 (3)	C24—H24B	0.9700
C6—C11	1.409 (3)	C25—C26	1.520 (3)
C7—C8	1.420 (4)	C25—H25A	0.9700
C7—H7	0.9300	C25—H25B	0.9700
C8—C9	1.368 (4)	C26—C27	1.510 (3)
C8—H8	0.9300	C26—H26A	0.9700
C9—C10	1.407 (4)	C26—H26B	0.9700
C9—H9	0.9300	C27—O4	1.4372 (19)
C10—C11	1.412 (2)	C27—H27A	0.9700
C10—C15	1.418 (4)	C27—H27B	0.9700
C11—C12	1.399 (2)	C28—O4	1.3640 (19)
C12—C13	1.365 (2)	C28—C29	1.388 (2)
C13—C14	1.412 (3)	C28—C33	1.405 (2)
C13—H13	0.9300	C29—C30	1.378 (3)
C14—C15	1.345 (4)	C29—H29	0.9300
C14—H14	0.9300	C30—C31	1.369 (3)
C15—H15	0.9300	C30—H30	0.9300
C16—N1	1.456 (2)	C31—C32	1.385 (3)
C16—H16A	0.9600	C31—H31	0.9300
C16—H16B	0.9600	C32—C33	1.389 (2)
C16—H16C	0.9600	C32—H32	0.9300
C17—O2	1.2165 (17)		
			
N1—C1—C2	105.89 (12)	C19—C18—C23	118.57 (14)
N1—C1—H1A	110.6	C19—C18—C17	117.88 (14)
C2—C1—H1A	110.6	C23—C18—C17	123.51 (13)
N1—C1—H1B	110.6	C20—C19—C18	121.12 (17)
C2—C1—H1B	110.6	C20—C19—H19	119.4
H1A—C1—H1B	108.7	C18—C19—H19	119.4
C33—C2—C3	115.53 (12)	C21—C20—C19	119.44 (17)
C33—C2—C1	117.14 (13)	C21—C20—H20	120.3
C3—C2—C1	102.89 (12)	C19—C20—H20	120.3
C33—C2—H2	106.9	C20—C21—C22	120.97 (17)
C3—C2—H2	106.9	C20—C21—H21	119.5
C1—C2—H2	106.9	C22—C21—H21	119.5
C17—C3—C2	115.55 (12)	C21—C22—C23	120.21 (17)
C17—C3—C4	112.42 (11)	C21—C22—H22	119.9
C2—C3—C4	101.87 (11)	C23—C22—H22	119.9
C17—C3—H3	108.9	O3—C23—C22	123.56 (14)
C2—C3—H3	108.9	O3—C23—C18	116.75 (12)
C4—C3—H3	108.9	C22—C23—C18	119.62 (14)
N1—C4—C12	116.98 (12)	O3—C24—C25	106.31 (13)
N1—C4—C3	99.72 (11)	O3—C24—H24A	110.5
C12—C4—C3	114.99 (12)	C25—C24—H24A	110.5
N1—C4—C5	111.72 (12)	O3—C24—H24B	110.5
C12—C4—C5	102.82 (12)	C25—C24—H24B	110.5
C3—C4—C5	110.94 (12)	H24A—C24—H24B	108.7
O1—C5—C6	128.49 (16)	C24—C25—C26	113.63 (16)
O1—C5—C4	124.00 (14)	C24—C25—H25A	108.8
C6—C5—C4	107.52 (13)	C26—C25—H25A	108.8
C7—C6—C11	120.19 (18)	C24—C25—H25B	108.8
C7—C6—C5	132.5 (2)	C26—C25—H25B	108.8
C11—C6—C5	107.26 (15)	H25A—C25—H25B	107.7
C6—C7—C8	117.2 (3)	C27—C26—C25	114.63 (16)
C6—C7—H7	121.4	C27—C26—H26A	108.6
C8—C7—H7	121.4	C25—C26—H26A	108.6
C9—C8—C7	122.8 (2)	C27—C26—H26B	108.6
C9—C8—H8	118.6	C25—C26—H26B	108.6
C7—C8—H8	118.6	H26A—C26—H26B	107.6
C8—C9—C10	121.2 (2)	O4—C27—C26	109.63 (13)
C8—C9—H9	119.4	O4—C27—H27A	109.7
C10—C9—H9	119.4	C26—C27—H27A	109.7
C9—C10—C11	115.6 (2)	O4—C27—H27B	109.7
C9—C10—C15	128.0 (2)	C26—C27—H27B	109.7
C11—C10—C15	116.4 (2)	H27A—C27—H27B	108.2
C12—C11—C6	113.94 (14)	O4—C28—C29	125.17 (15)
C12—C11—C10	123.1 (2)	O4—C28—C33	114.55 (14)
C6—C11—C10	122.95 (19)	C29—C28—C33	120.27 (16)
C13—C12—C11	118.37 (16)	C30—C29—C28	120.15 (18)
C13—C12—C4	133.39 (16)	C30—C29—H29	119.9
C11—C12—C4	108.24 (14)	C28—C29—H29	119.9
C12—C13—C14	119.3 (2)	C31—C30—C29	120.66 (18)
C12—C13—H13	120.3	C31—C30—H30	119.7
C14—C13—H13	120.3	C29—C30—H30	119.7
C15—C14—C13	122.7 (2)	C30—C31—C32	119.37 (18)
C15—C14—H14	118.6	C30—C31—H31	120.3
C13—C14—H14	118.6	C32—C31—H31	120.3
C14—C15—C10	120.09 (19)	C31—C32—C33	121.86 (18)
C14—C15—H15	120.0	C31—C32—H32	119.1
C10—C15—H15	120.0	C33—C32—H32	119.1
N1—C16—H16A	109.5	C32—C33—C28	117.69 (16)
N1—C16—H16B	109.5	C32—C33—C2	119.58 (15)
H16A—C16—H16B	109.5	C28—C33—C2	122.73 (14)
N1—C16—H16C	109.5	C16—N1—C4	115.89 (14)
H16A—C16—H16C	109.5	C16—N1—C1	115.79 (15)
H16B—C16—H16C	109.5	C4—N1—C1	108.04 (12)
O2—C17—C18	119.55 (13)	C23—O3—C24	119.06 (12)
O2—C17—C3	121.26 (13)	C28—O4—C27	123.29 (13)
C18—C17—C3	119.01 (12)		
			
N1—C1—C2—C33	−136.90 (13)	C2—C3—C17—O2	−22.5 (2)
N1—C1—C2—C3	−9.01 (15)	C4—C3—C17—O2	93.80 (17)
C33—C2—C3—C17	−75.61 (16)	C2—C3—C17—C18	162.43 (12)
C1—C2—C3—C17	155.49 (12)	C4—C3—C17—C18	−81.22 (15)
C33—C2—C3—C4	162.22 (12)	O2—C17—C18—C19	−45.8 (2)
C1—C2—C3—C4	33.32 (14)	C3—C17—C18—C19	129.27 (15)
C17—C3—C4—N1	−169.82 (11)	O2—C17—C18—C23	131.81 (16)
C2—C3—C4—N1	−45.52 (13)	C3—C17—C18—C23	−53.1 (2)
C17—C3—C4—C12	−43.82 (17)	C23—C18—C19—C20	−0.4 (2)
C2—C3—C4—C12	80.48 (14)	C17—C18—C19—C20	177.41 (16)
C17—C3—C4—C5	72.31 (14)	C18—C19—C20—C21	−1.7 (3)
C2—C3—C4—C5	−163.39 (11)	C19—C20—C21—C22	1.7 (3)
N1—C4—C5—O1	−49.2 (2)	C20—C21—C22—C23	0.4 (3)
C12—C4—C5—O1	−175.47 (16)	C21—C22—C23—O3	−179.41 (15)
C3—C4—C5—O1	61.1 (2)	C21—C22—C23—C18	−2.5 (2)
N1—C4—C5—C6	130.97 (13)	C19—C18—C23—O3	179.58 (13)
C12—C4—C5—C6	4.71 (15)	C17—C18—C23—O3	1.9 (2)
C3—C4—C5—C6	−118.73 (13)	C19—C18—C23—C22	2.5 (2)
O1—C5—C6—C7	−4.9 (3)	C17—C18—C23—C22	−175.18 (14)
C4—C5—C6—C7	175.0 (2)	O3—C24—C25—C26	71.99 (18)
O1—C5—C6—C11	175.99 (17)	C24—C25—C26—C27	−135.12 (16)
C4—C5—C6—C11	−4.20 (17)	C25—C26—C27—O4	74.2 (2)
C11—C6—C7—C8	−0.7 (3)	O4—C28—C29—C30	−178.10 (15)
C5—C6—C7—C8	−179.8 (2)	C33—C28—C29—C30	0.5 (3)
C6—C7—C8—C9	−1.7 (4)	C28—C29—C30—C31	0.1 (3)
C7—C8—C9—C10	2.2 (4)	C29—C30—C31—C32	−0.5 (3)
C8—C9—C10—C11	−0.1 (3)	C30—C31—C32—C33	0.2 (3)
C8—C9—C10—C15	179.2 (2)	C31—C32—C33—C28	0.4 (2)
C7—C6—C11—C12	−177.33 (17)	C31—C32—C33—C2	−178.72 (16)
C5—C6—C11—C12	1.9 (2)	O4—C28—C33—C32	178.01 (14)
C7—C6—C11—C10	2.8 (3)	C29—C28—C33—C32	−0.8 (2)
C5—C6—C11—C10	−177.89 (16)	O4—C28—C33—C2	−2.9 (2)
C9—C10—C11—C12	177.83 (18)	C29—C28—C33—C2	178.32 (15)
C15—C10—C11—C12	−1.6 (3)	C3—C2—C33—C32	117.19 (16)
C9—C10—C11—C6	−2.3 (3)	C1—C2—C33—C32	−121.30 (16)
C15—C10—C11—C6	178.22 (17)	C3—C2—C33—C28	−61.87 (19)
C6—C11—C12—C13	−178.50 (15)	C1—C2—C33—C28	59.65 (19)
C10—C11—C12—C13	1.3 (3)	C12—C4—N1—C16	48.6 (2)
C6—C11—C12—C4	1.21 (19)	C3—C4—N1—C16	173.22 (14)
C10—C11—C12—C4	−178.95 (15)	C5—C4—N1—C16	−69.49 (17)
N1—C4—C12—C13	53.2 (2)	C12—C4—N1—C1	−83.20 (16)
C3—C4—C12—C13	−63.3 (2)	C3—C4—N1—C1	41.42 (14)
C5—C4—C12—C13	176.04 (18)	C5—C4—N1—C1	158.71 (12)
N1—C4—C12—C11	−126.41 (15)	C2—C1—N1—C16	−152.69 (14)
C3—C4—C12—C11	117.08 (14)	C2—C1—N1—C4	−20.84 (16)
C5—C4—C12—C11	−3.61 (15)	C22—C23—O3—C24	−1.9 (2)
C11—C12—C13—C14	0.0 (3)	C18—C23—O3—C24	−178.90 (14)
C4—C12—C13—C14	−179.62 (17)	C25—C24—O3—C23	−178.90 (14)
C12—C13—C14—C15	−1.0 (3)	C29—C28—O4—C27	−8.9 (2)
C13—C14—C15—C10	0.7 (3)	C33—C28—O4—C27	172.38 (13)
C9—C10—C15—C14	−178.8 (2)	C26—C27—O4—C28	−107.64 (17)
C11—C10—C15—C14	0.5 (3)		

### Hydrogen-bond geometry (Å, º)

**Table:**

*D*—H···*A*	*D*—H	H···*A*	*D*···*A*	*D*—H···*A*
C9—H9···N1^i^	0.93	2.62	3.535 (3)	167
C15—H15···O1^i^	0.93	2.50	3.414 (2)	168
C27—H27*B*···O2^ii^	0.97	2.48	3.403 (2)	158
C29—H29···O2^ii^	0.93	2.57	3.450 (2)	159

Symmetry codes: (i) −*x*, *y*−1/2, −*z*+1/2; (ii) −*x*+1, *y*+1/2, −*z*+1/2.
